# Prevalence of *HER2* Expression and Its Correlation with Clinicopathological Parameters in Gastric or Gastroesophageal Junction Adenocarcinoma in North-East Indian Population 

**DOI:** 10.31557/APJCP.2019.20.4.1139

**Published:** 2019

**Authors:** Partha S Roy, Tomar Nyodu, Munlima Hazarika, B J Saikia, C Bhuyan, Amit Inamdar, C W Nyuthe, B Borthakur, J D Sharma

**Affiliations:** 1 *Department of Medical Oncology, *; 2 *Department of Surgical Oncology, *; 3 *Department of Pathology, Dr B Borooah Cancer Institute, Guwahati, Assam, India. *

**Keywords:** Gastric cancer, gastroesophageal adenocarcinoma, human epidermal factor receptor, immunohistochemistry

## Abstract

**Objective:**

Human epidermal growth factor receptor 2 (*erbb2/HER2*) overexpression, has now been implicated in advanced gastric and gastroesophageal junction cancers. The study was conducted to determine the rate of *HER2 *positivity in patients with locally advanced or metastatic gastric and gastroesophageal adenocarcinoma in North-East India and to assess the impact of various demographic and clinical parameters on *HER2* positivity.

**Methods:**

A total of 68 patients of age >18 years of gastric and gastroesophageal adenocarcinoma diagnosed on histopathological examination from September 2016 to February 2018 at Dr B Borooah Cancer Institute, Assam were enrolled for the observational (epidemiological) study. All patients were subjected to the *HER2* immunohistochemistry test using a FDA-approved, standardized test kit. *HER2 *expression was correlated with various demographic and clinicopathological parameters.

**Results:**

The overall rate of HER2 positivity in the population studied was 56% (n=38). The rate was non-significantly higher in male, older age group (>60 years) and Hindu population. Similarly,* HER2* positivity rate was higher in patients with well differentiated histology and was more common in patients with stage II and III diseases, but neither of the associations is statistically significant. *HER2* positivity rate was significantly higher in proximal and in GEJ tumours (56% versus 44%, P=0.002).

**Conclusion:**

*HER2* overexpression was evident in 56% of the North-East Indian patients with locally advanced and metastatic gastric and gastroesophageal adenocarcinoma. The overexpression correlated significantly with primary tumour site. Routine testing of gastric and gastroesophageal tumours for *HER2* expression is recommended to provide a therapeutic advantage in Indian patients.

## Introduction

Gastric cancer is a leading health-care problem worldwide and is the fifth most common cancer in the world, with 952,000 new cases diagnosed in 2012 Worldwide 0.7 million people die of gastric cancer each year (accounts for 8.8% of all cancer-related deaths) [GLOBACON, 2012]. Gastric cancer is the second most common cancer among men and third-most among females in Asia (Rahman et al., 2014) and is the second most common cause of cancer-related deaths in India (Dikshit et al., 2012). A high incidence of gastric cancer has been reported from Southeast Asia, most commonly from Japan, China and South Korea (Ferlay et al., 2012). 

The age-adjusted rate (AAR) of gastric cancer among urban registries in India is (3.0-13.2) compared to the worldwide AAR (4.1-95.5) with China having the highest incidence of AAR. Amongst all the population based cancer registries in India, Papumpure district (Arunachal Pradesh, India) has the highest AAR of 50.2% followed by Aizawl district (Mizoram, India) with AAR of 43.9% both in males and females (ICMR 2016). The long term ingestion of high concentration of nitrates found in dried, smoked and salted foods appears to be associated with a higher risk in this part of the world. 

In advanced stage disease, the 5-year survival is less than 30% in developed countries and is around 20% in developing countries (Kang et al., 2010). Surgery is the mainstay of treatment in early stage disease. Although chemotherapy improves survival in patients with advanced gastric cancer, there is no globally accepted standard first-line regimen. Even with optimal combination chemotherapy, the median survival in Western studies remains less than 1 year in advanced settings (Van Cutsem et al., 2006; Cunningham et al., 2008). Attention has turned to potential molecular targets in gastric and gastroesophageal junction (GEJ) adenocarcinomas.

Human epidermal growth factor receptor 2 (*HER2*) is a 185-kDa trans-membrane tyrosine kinase receptor of the HER family and is a proto-oncogene encoded by erb2 on chromosome 17. *HER2 *gene amplification and protein overexpression play an important role in the proliferation, apoptosis, adhesion, angiogenesis and aggressiveness of many solid tumours. Although *HER2 *expression was initially associated with breast cancer it has now been implicated in various cancers including, colon, bladder, ovary, uterine cervix, gastric and GEJ cancers (Reicheelt et al., 2007; Kaur et al., 2011). 

*HER2 *overexpression in gastric cancer IHC was first described in 1986 (Gravaalos et al., 2008). Large numbers of studies on *HER2 *positivity in gastric cancer have been published from Western world, relating it to important clinicopathological characteristics and survival. *HER2 *positivity in gastric cancer ranges from as low as 4% to as high as 53% (Kunz et al., 2012; Shan et al., 2013; Abrahao-Machad et al., 2016; Gharsalli et al., 2017). In a literature review of a large population involving more than 8000 patients, median *HER2 *positivity of 20.2% was observed (Maressch et al., 2012). The *HER2 *expression rate in Asians may be higher than that in Europeans (Lei et al., 2017). In an Egyptian population of 76 resectable gastric carcinoma patients, the rate of HER2/neu positivity was found to be high (about 54%) (Abdel-Salam et al., 2018). 

Routinely one paraffin block is used for HER2/neu assessment by IHC. Using two paraffin blocks to assess HER2/neu expression may identify more patients with HER2/neu positive gastric cancer, by reducing false-negative rate (Xiaowen et al., 2015). 

*HER2 *is a well-established therapeutic target in breast cancer. *HER2 *overexpression in metastatic gastric cancer patients may be a predictor of poor prognosis (Aguair Junior et al., 2016). Addition of trastuzumab to chemotherapy has been shown to improve the survival in patients with HER2-positive gastric and GEJ carcinoma in Trastuzumab for Gastric Cancer (ToGA) trial (Bang et al., 2010). In 2010 trastuzumab received approval for the use in combination with 5-flurouracil (5-FU) or capecitabine plus cislatin for the first-line treatment of patients with *HER2 *positive metastatic adenocarcinoma of the gastric or GEJ after the result of the phase III ToGA trial (Bilici et al., 2014). Median overall survival was significantly improved with trastuzumab plus chemotherapy compared to chemotherapy alone: 13.8 (12.0-16.0) months versus 11.1 (10.0-13.0) months, respectively (P = 0.0046) (Bang et al., 2010).

Studies conducted so far suggest the need for optimizing *HER2 *testing as appropriate interpretation for these test results could translate into delivery of optimal therapy. It has been reported that only patients with high levels of *HER2 *expression derive maximum benefit from trastuzumab based therapy (Ruschoff et al., 2012).

In the year 2015, India reported a total of 63,043 gastric cancer cases, with 3,806 cases from North-East India. Dr B Borooah Cancer Institute, a regional cancer centre of the North-East India recorded 320 cases of gastric adenocarcinoma in 2015 (ICMR, 2016). 

However, studies on the frequency of HER2-positivity among the patients in the North-East region of the India are limited. Therefore, this study was conducted to evaluate the prevalence of *HER2 *in gastric and GEJ adenocarcinoma in North-East Indian population and to correlate *HER2 *overexpression with clinicopathological parameters.

## Materials and Methods

It was an observational study (epidemiological study) wherein the data were collected in a prospective manner. A total of 340 cases of gastric and GEJ adenocarcinoma diagnosed on biopsy specimen in the pathology department of Dr B Borooah Cancer Institute (BBCI) over a time span of one and half years (September 2016 to February 2018) were included in the study. The study received the approval from the Institutional Ethics Committee (BBCI/M-119/MEC/2195/2016).

The study included men and women over 18 years of age, newly diagnosed with locally advanced or metastatic histopathologically confirmed gastric/GEJ adenocarcinoma. Patients aged less than 18 years and patients who received chemotherapy and/or radiotherapy earlier were excluded from the study.

The primary objective of the study was to determine the incidence of *HER2 *positivity in patients with locally advanced or metastatic gastric/GEJ adenocarcinoma in North-East India. The secondary objective was to evaluate correlation of *HER2 *overexpression with demographic and clinicopathological parameters.

**Table 1:** Human Epidermal Growth Factor Receptor 2 Immunohistochemistry Scoring Criteria for Gastric Cancer (Hofmann et al.)

Staining pattern	IHC score	HER2 overexpression
No reactivity or membranous reactivity in < 10% of tumour cells	0	Negative
Faint or barely perceptible reactivity in ≥10% of tumour cells; cells are reactive only in part of their membrane	1+	Negative
Weak to moderate, complete, basolateral, or lateral membranous reactivity in ≥10% of tumour cells	2+	Equivocal*
Strong, complete, basolateral, or lateral membranous reactivity in ≥10% of tumour cells	3+	Positive

*Equivocal HER2 expression by IHC to be confirmed by FISH

**Table 2:** Association between *HER2* Status and Clinicopathological Parameters

	Overall n = 68 (%)	HER2 positive n = 38 (55.8%)	HER2 negative n = 30 (44.1%)	P value
Age Group (years)				0.09
<40	5 (7.3)	3 (7.8)	2 (6.6)	
40 – 60	27 (54.4)	21 (55.0)	16 (53.3)	
>60	26 (38.2)	14 (36.8)	12 (40.0)	
Gender				0.189
Male	40 (58.8)	25 (65.7)	15 (50.0)	
Female	28 (41.1)	13 (34.2)	15 (50.0)	
Race				0.094
Hindu	37 (54.4)	25 (65.7)	12 (40.0)	
Muslim	25 (36.7)	11 (28.9)	14 (46.6)	
Christian	6 (8.8)	2 (5.2)	4 (13.3)	
Tumour Location				0.002
GEJ	14 (20.5)	8 (21.0)	6 (20.0)	
Proximal (excluding GEJ)	12 (17.6)	13 (34.2)	0 ( - )	
Body/distal gastric	42 (61.7)	17 (44.7)	24 (80.0)	
Histological Grade				0.051
Well differentiated	23 (33.8)	19 (50.0)	4 (13.3)	
Moderately differentiated	20 (29.4)	10 (26.3)	10 (33.3)	
Poorly differentiated	25 (36.7)	9 (23.6)	14 (53.3)	
TNM Staging (clinical)				0.312
Stage II	10 (14.7)	6 (15.7)	4 (13.3)	
Stage III	29 (42.6)	16 (42.1)	13 (43.3)	
Stage IV	22 (32.3)	10 (26.3)	12 (40.0)	
NK	7 (10.2)	6 (15.7)	1 (3.3)	

GEJ, gastroesophageal junction; NK, stage not known)

The pathology records of all patients diagnosed with gastric/GEJ adenocarcinoma at BBCI were obtained electronically from September 2016 to February 2018 and investigations for *HER2 *status were included in the study. Among 340 histopathologically confirmed cases of gastric/GEJ adenocarcinoma, 68 cases that were subjected to further *HER2 *immunohistochemistry (IHC) test were included for the final analysis. 

Clinical data, including patient’s demographic profile and pathological parameters namely, tumour site, stage and histological grade were recorded.

*HER2 *overexpression was determined by IHC test using an FDA-approved, standardised test kit (Hercep test kit^TM^, DakoCytomation Denmark A/S^TM^, Glostrup, Denmark). For determination of *HER2 *expression, a semi-quantitative scoring criterion was applied in sections and the results were recorded as positive, negative and equivocal according to the staining patterns (Hofmann et al., 2008; Abrahao-Machad et al., 2016). (Table 1) Scores of 3+ was taken as positive. Score values of 0 and 1+ were taken as negative. *HER2 *IHC score 2+ were considered as equivocal and should be further tested for gene amplification by fluorescence in-situ hybridization (FISH) method. Confirmation of cases reported as equivocal (*HER2 *IHC score 2+) by FISH could not be done in our study due to financial restraints. Therefore, score 2+ was also included as negative for all practical purposes. Overall *HER2 *overexpression in gastric and GEJ adenocarcinoma was evaluated and was further correlated with various clinicopathological features. Impact of trastuzumab on survival in HER2-positive cases was not studies.

Data that are collected was checked for consistency and completeness, and entered in data base software. Summarization and analysis was carried out using descriptive statistics. Statistical analysis was done with SPSS version 16.0 software. Categorical variables were expressed as frequencies and percentages. Nominal categorical data between the groups were compared using Chi-square test (χ^2^). Survival analysis was done using Kaplan-Meier (log-rank tests) survival curves. For all statistical tests, a P < 0.05 was considered as statistically significant (at 95% CI).

## Results

A total of 68 cases were included for the study. Clinicopathological characteristics and their association with *HER2 *final status and IHC scores are shown in Table 2.

*Patient demographics and tumour characteristics*

Patients age at diagnosis ranged from 32 to 80 years (mean age of presentation was 58 years). Of total 68 patients, 40 (59%) were male and 28 (41%) were female (male to female ratio was 1.4:1). The most common age group was 40 to 60 years (54.4%) followed by age group of more than 60 years (38%). The patient’s age less than 40 years constitutes only 7%. Majority of the patients are from Hindu community (54%) followed by Muslim community (37%). The location of the tumour was at the GEJ in 20.5%, proximal part of stomach (excluding GEJ) in 17.6%, and body/distal part of stomach in 61.7% patients. Poorly differentiated tumours were observed in 25 (36.7%), moderately differentiated tumour in 20 (29.4%), and well differentiated tumours in 23 (33.8%) of patients.

*HER2 *overexpression of 3+ was considered as positive; whereas score values of 2+, 1+ and 0 were taken as negative. Of the total 68 cases enrolled the study, 56% (n=38) tested positive for *HER2 *by IHC (score 3+) and 44% (n=30) were negative for *HER2 *(score 2+, 1+ and 0) by IHC.

The median age of presentation is 57.5 years in *HER2 *positive cases and is 58 years in *HER2 *negative cases. The maximum number of *HER2 *positivity tumours was detected in patients aged more than 40 years (40 to 60 years age group). None of the patients in the IHC3+ category were younger than 32 years of age and the oldest patient in the series was an 80 year old man, whereas the youngest age was 35 years in *HER2 *negative group. 

The rate of *HER2 *positivity was higher in men (66% versus 34%) than women, but there was no association between *HER2 *expression and patient’s gender.

*Association/correlation of HER2 status with clinicopathological features*

There was no statistically significant correlation between *HER2 *positivity with age, gender, race, and tumour staging.

*HER2 positivity rate and gastric cancer site*

Tumour located in different parts of the stomach such as the fundus, lesser curvature, body, antrum and pylorus were grouped as gastric body tumours, whereas tumour located in the cardia, lower end of esophagus, GEJ, proximal stomach, and cardio-esophageal junction were grouped as GEC (GEJ cancers) tumours. The incidence of *HER2 *positivity in GEC (55%) was higher than that in gastric body (45%); this association was found to be statistically significant (P=0.006). The overall *HER2 *positivity rate for patients characterised as having a gastric site (proximal and distal stomach combined) was 79% and 21% for those with GEJ site, where the association was found to highly significant (P=0.002).

Tumours arising in the distal stomach accounted for 45% of IHC3+ cases; 20.5% were located in the in the GEJ and another 34% located in the proximal stomach (excluding GEJ), where the variation seen to be statistically significant (P=0.002). In fact, this is the first report of *HER2 *positivity status in pure GEJ tumours, where GEJ tumours accounts for 20.5% (n=14) of total 68 case. When all the proximal tumours are combined (that also includes GEJ tumours), *HER2 *positivity seen in 55% of cases (variation is significant with P=0.006).

*HER2 positivity rate and tumour grade/tumour differentiation*

Our study showed a non-significant association between *HER2 *expression and well differentiated histology. The *HER2 *positivity rate for tumour grade was 50% for well differentiated, 24% for poorly differentiated, and 26% for moderately differentiated (P=0.051). Studies have shown both an association and non-association between *HER2 *overexpression and tumour differentiation. This discrepancy may be attributed to varying sample sizes and lower prevalence of *HER2 *in gastric/gastroesophageal cancers. .

*HER2 *negative carcinomas with *HER2 *IHC score 1+ and 0 aggregated all tumours with poorly differentiated histological grades. Poorly differentiated histology accounts for 47% of all *HER2 *negative carcinomas (score 2+, 1+ and 0), whereas, poorly differentiated histology is seen in only 29% of *HER2 *positive tumours. *HER2 *positive tumours are more associated with well differentiated histology (in 50% cases versus in 20% of *HER2 *negative cases); however, the association is statistically not significant (P=0.051).

*HER2 positivity rate and TNM staging*

HER2-positivity was more commonly found to be associated with stage III diseases (16% in stage II, 42% in stage III and 26% in stage IV). However, no significant association was observed between *HER2 *expression and TNM staging.

*Statistical significance of HER2 positivity rates between subgroups*

The chi-squared (χ^2^) test was used to assess the statistical significance of *HER2 *positivity rates across the different demographic parameters for the following subgroups: gastric cancer site, age and tumour grade. For all analyses done, the (χ^2^) test was significant, for the association between tumour location and *HER2 *expression concluding that there was at least one significant difference between the *HER2 *positive rates and the demographic parameters of the individual subgroups.

## Discussion

*HER2 *expression in gastric/gastroesophageal cancers has been known for over years. In addition to being implicated in the pathogenesis of cancers, *HER2 *has also been evaluated as a therapeutic target. Efficacy of trastuzumab in patients with breast cancer has led to emerging interest in its antitumor activity in patients with *HER2 *positive gastric carcinoma. Many studies have investigated the role of trastuzumab in the management of gastric/gastroesophageal cancer. The implication of *HER2 *overexpression on prognosis remains unknown in patients with metastatic gastric cancer who do not receive trastuzumab. 

Our study describes findings from the clinical audit of *HER2 *expression in gastric cancer in North-East India centre, providing the first insight into the rate of HER2-positivity in gastric and gastroesophageal adenocarcinoma. Based on an analysis of 340 cases of gastric/gastroesophageal cancer biopsy specimens, 68 cases were finally analysed for *HER2 *expression by IHC. An overall *HER2 *positivity rate of 56% was observed in our study. The rate is higher compared to the range reported in the literature from different populations (ranges from as low as 4.4% to 53.4%) (Abdel-Salam et al., 2018).

In our study, *HER2 *overexpression was observed in 56% cases of gastric or gastroesophageal adenocarcinoma which is the highest positivity rate reported till date. Variation in HER2-positivity rates between countries and different populations may be explained in part by the proportion of carcinomas with certain characteristics with which HER2-positivity has been correlated. HER2-positivity rates were higher in the proximal gastric cancer site than in the gastric site (55% versus 44%), a correlation that has been consistently reported in the literature (Shan et al., 2013; Van et al., 2015). Higher *HER2 *expression was seen in older age, males and in Hindu population, however, the correlation was not statistically significant. Similarly, in an Iranian population, older age and male sex appears to be more associated with HER2/neu gene expression (Azarhoosh et al., 2017). A non-significant association was also noted between *HER2 *overexpression and stage II/III gastric/gastroesophageal cancers. 

*HER2 *overexpression was correlated with the various clinicopathological parameters in gastric cancer patients: male sex, proximally located tumour, advanced stage, lymph node metastasis, distant metastasis, poorly differentiated tumour, and intestinal subtype (Lei et al., 2017). However, in a small case series from Pakistan, HER2/neu was significantly expressed in low grade gastric cancer, predominantly seen in females, age >60 years, intestinal subtype, and stage IIIC tumours (Shabbir et al., 2018).

HER2-positivity was associated with intestinal subtype (P=0.048), well to moderately differentiated tumour (P=0.004) and presence of lymphovascular invasion (P=0.031) (Renato et al., 2015). In our study we did not see any significant correlation between *HER2 *overexpression and histological grades of tumour, although a trend towards significance is observed with well differentiated histology being more commonly seen in *HER2 *positive cases (P=0.051). However, *HER2 *IHC score 1+ and 0 were exclusively seen to be associated with poorly differentiated histology.

Consistent with previous reports, HER2-positivity was more frequent with well- or moderately differentiated histology (50% and 26%, respectively) (Shan et al., 2013; Son et al., 2014; Rajagopal et at., 2015), compared with poorly differentiated carcinomas (23%). In our study, HER2/neu gene expression is more commonly seen in stage III disease (42% in stage III versus 26% in stage IV), unlike other studies where HER2/neu gene expression is significantly more seen in stage IV of gastric cancer with a larger tumour mass, that can be partly explained by slightly higher numbers of patients with stage III disease (Azarhoosh et al., 2017). A study from South Asian population also found significantly higher HER2/neu expression in low grade tumours and stage III tumours (Shabbir et al., 2018).

Although, many authors did not report any significant association between site of tumour and HER2-positivity, the conflicting results have been reported regarding tumour location and *HER2 *expression. In our study proximally located tumours constitutes 38% of cases which may be the reason for higher *HER2 *positivity rate of 55% (P=0.006). GEJ adenocarcinoma alone accounts for 21% (n=14) of all cases (with *HER2 *positivity of 21%); proximal tumours other than GEJ tumours accounts for 18% (*HER2 *positivity of 34%), which is highly significant (P=0.002). Fan et al., (2013) has also reported a significant association of *HER2 *positivity with proximal tumours. In fact, this is the first report of *HER2 *positivity status in pure GEJ adenocarcinoma. Another reason for high *HER2 *positivity rate may be because of different demographic features of the North-East part of the India as compared to other part of the World. There was no previous similar study available to compare our study in the North-East part of the India. 

A number of limitations of this study warrant mention. Most notably, a considerable amount of patient data were not collected particularly on tumour stage and gender, preventing further analysis of these parameters. The main weakness of our study was the non-availability of FISH/ISH analysis to determine *HER2 *amplification for *HER2 *equivocal (score 2+) cases, which could have probably influenced the variations/associations amongst the different clinicopathological parameters. Although the IHC continues to play an essential role in *HER2 *status assessment, the overall reliability of *HER2 *evaluation by IHC, however, can be affected by diverse pre-analytical, analytical and post-analytical variables.

The high proportion of equivocal (IHC 2+) *HER2 *cases is also a concern. Many patients in Asian countries are not referred for confirmatory FISH/ISH testing, primarily due to the high cost of these assays and *HER2 *targeted therapy should a positive result be found. 

Given these limitations it is probably unwise to consider these results as a wholly accurate representation of the incidence of HER2-positivity in gastric/gastroesophageal cancer in North-East India. A well conducted prospective randomized trial probably can give us the exact prevalence of *HER2 *positivity in this unique geographical area of North-East India.

In conclusion, this study highlights the need of further defining the role of *HER2 *expression in gastric/gastroesophageal adenocarcinoma in North-East Indian patients. Given the significant burden of gastric cancer in this region, these data are important to increase our understanding and awareness of the disease. Increased education and awareness of *HER2 *testing in gastric/gastroesophageal cancer, as well as improved early detection are what is needed to improve outcomes. Routine testing of gastric and gastroesophageal tumours for *HER2 *expression is recommended to provide a therapeutic advantage in Indian patients.

## Funding source

None.

## Conflict of interest

The authors have no conflicts of interest to declare.
